# A *WUSCHEL*-like homeobox gene, *OsWOX3B* responses to *NUDA/GL-1* locus in rice

**DOI:** 10.1186/1939-8433-5-30

**Published:** 2012-10-03

**Authors:** Honglei Zhang, Kun Wu, Yufeng Wang, Yu Peng, Fengyi Hu, Lu Wen, Bin Han, Qian Qian, Sheng Teng

**Affiliations:** 1 Shanghai Institute of Plant Physiology and Ecology, Shanghai Institute for Biological Sciences, The Chinese Academy of Sciences, Shanghai, 200032 China; 2 State Key Laboratory of Rice Biology, China National Rice Research Institute, Chinese Academy of Agricultural Sciences, Hangzhou, 310006 China; 3 Institute of Food Crops, Yunnan Academy of Agricultural Sciences, Kunming, 650205 China; 4 Puer Agricultural Research Institute, Puer, 66500 China

**Keywords:** Yunnan nuda rice, Nuda/glabrous, Map-based cloning, *WUSCHEL*-like homeobox gene *OsWOX3B*

## Abstract

**Background:**

Most of the rice varieties are pubescent. However, the presence of trichomes is an undesirable characteristic in rice production because trichomes can cause atmospheric pollution. The use of glabrous rice varieties represents a solution to this problem. Yunnan Nuda Rice, a glabrous cultivar that constitutes approximately 20% of rice germplasms in Yunnan can provide important recourse for breeding of glabrous rice varieties.

**Results:**

The “Nuda” phenotype in Yunnan Nuda Rice was found to be controlled by a single recessive allelic gene within the well-characterized *GL-1* locus. A high-resolution genetic and physical map was constructed using 1,192 Nuda individuals from the F_2_ population that was delivered from the cross between the Yunnan Nuda variety HMK and the pubescent TN1 variety. The *NUDA/GL-1* gene was mapped to a 28.5 kb region containing six annotated genes based on the Nipponbare genomic sequence. By comparing the sequences and expression patterns of different pubescent and glabrous varieties, *LOC_Os05g02730*, a *WUSCHEL*-like homeobox gene (*OsWOX3B*) was identified as the candidate gene. This hypothesis was confirmed by RNA interference (RNAi) and transgenic complementation. Trichome deficiency in RNAi lines was associated with increased efficiency of grain packaging but did not affect the main agronomic traits.

**Conclusion:**

*NUDA/GL-1* locus encodes *OsWOX3B* gene.

**Electronic supplementary material:**

The online version of this article (doi:10.1186/1939-8433-5-30) contains supplementary material, which is available to authorized users.

## Background

Trichomes are ubiquitous in land plants (Southwood[1986]; Werker[2000]). The density, morphology, and chemical composition of leaf hairs vary widely and these factors contribute to their diverse physiological, physical and chemical functions ([Southwood 1986];[Werker 2000]). However, the presence of trichomes is an undesirable characteristic in rice production due to the generation of dust during harvesting and grain manipulating processes. Trichomes are important causes of atmospheric pollution and these pollutants can cause acute and chronic irritation of the eyes, skin, and respiratory tract, which subsequently result in intolerable itching and allergy, and even longstanding diseases such as Rice Miller’s Syndrome (Lim et al.[1984]). Therefore, the use of glabrous rice varieties, which are trichome-deficient on the leaves and glumes, represents a solution to this problem.

Natural and artificial glabrous mutants have been identified in model plants such as *Arabidopsis* ([Karkkainen and Agren 2002]) and cereal crop species including rice ([Foster and Rutger 1978]), maize (Moose et al.[2004]), wheat ([Leisle 1974]) barley ([Sato and Takeda 1992]) oats ([Sarkarung and Collins 1977]) pearl millet ([Kumar and Andrews 1993]) and sorghum ([Gibson and Maiti 1983]). In particular, trichome formation in *Arabidopsis* has been used as a model system to study various developmental and cellular mechanisms in plants ([Hulskamp 2004]; Marks[1997]). With mutagenesis screens, dozens of genes involved in trichome initiation, spacing, and shape have been identified in Arabidopsis ([Marks 1997];[Hulskamp and Schnittger 1998][Serna and Martin 2006] Szymanski et al.[2000]). These genes encode several classes of transcriptional factors, including MYB (*GL1*) (Larkin et al.[1993]; Oppenheimer et al.[1991]), WD-40 (*TTG1*) (Walker et al.[1999]), bHLH (*GL3/EGL3*) (Payne et al.[2000]) and HD-ZIP (*GL2*) (Johnson et al.[2002]; Szymanski et al.[2000]). Recent studies of *Arabidopsis* have shown a complex that consists of GL1-GL3/EGL3-TTG1 promote GL2 expression to regulate hair cell differentiation (Payne et al.[2000]; Lloyd et al.[2000]). Several other MYB proteins, including CYC, TRY (Schellmann et al.[2002]) and ETC1 (Kirik et al.[2004]), compete with GL1 by interacting with bHLH proteins to repress trichome initiation on the leaf (Schellmann et al.[2002]). However, no glabrous genes have been cloned in cereal crop species to date.

Glabrous rice varieties have been identified in different locations worldwide including America, Yunnan in China and regions of south-eastern and southern Asia. Two loci (*GL-1* and *GL-2*) for glabrous leaf and hull characteristics have been identified by classic genetic analysis. Two complementary genes (*Hla* and *Hlb*) have been identified for long pubescence on leaves, and one gene (*Hg*) has been identified for long pubescence on floral hulls (Nagao et al.[1960]). Among these genes, only the *GL-1* gene from American glabrous rice has been mapped to chromosome 5 with the use of restriction fragment length polymorphism (RFLP) markers (Yu et al.[1995]). This locus was further narrowed to a region of approximately 157 kb (Wang et al.[2009]) and 54 kb (Li et al.[2010]) by two independent research groups.

Yunnan is a center of rice landrace diversity. It is reported that about 20% of the Yunnan landraces are glabrous rice varieties, known as Yunnan Nuda Rice (Qin et al.[1997]). In this study, the glabrous phenotypes of Yunnan upland Nuda rice landrace HMK were characterized. By genetic analysis, the glabrous phenotype of HMK was shown to be conferred by a pair of recessive genes, and this locus was allelic with the reported *GL-1* locus. Furthermore, the *NUDA/GL1* gene was cloned using a map-based approach and identified as a *WUSCHEL*-like gene (*LOC_Os05g02730*) belonging to the homeodomain (HD) subfamily.

## Results

### Characterization of Yunnan nuda rice HMK variety

The presence of trichomes is ubiquitous in rice varieties. Leaf and grain phenotypes of the pubescent TN1 variety and the glabrous Yunnan Nuda Rice HMK variety were examined using stereomicroscopy and scanning electron microscopy (SEM).

**Figure 1:**
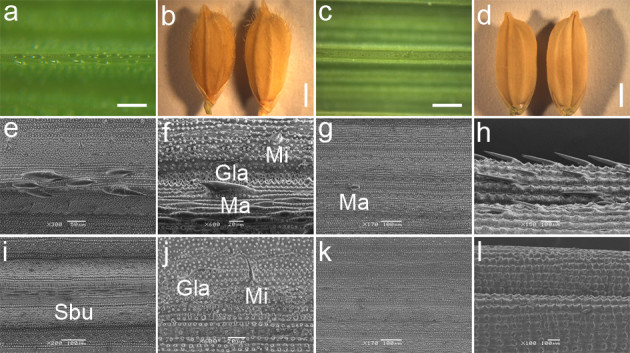
**Leaf and grain phenotypes of pubescent variety TN1 and glabrous Yunnan Nuda rice variety, HMK.** **a** TN1 leaf adaxial epidermis morphology (Scale bar: 0.5mm). **b** TN1 grain morphology (Scale bar: 1.5mm). **c** HMK leaf adaxial epidermis morphology (Scale bar: 0.5mm). **d** HMK grain morphology (Scale bar: 1.5mm). **e**, **f** Scanning electron microscope (SEM) images of TN1 leaf adaxial epidermis. **g** SEM images of TN1 leaf abaxial epidermis. **h** SEM images of TN1 grain epidermis. **i**, **j** SEM images of HMK leaf adaxial epidermis. **k** SEM images of HMK leaf abaxial epidermis. **l** SEM images of HMK grain epidermis. Ma, macro hairs; Mi, micro hairs; Gla, glandular hairs; Sbu, small bulges.

Trichomes were observed on the upper (adaxial) surface and along the veins of the leaf blade and on glumes of pubescent rice varieties, such as TN1 (Figures1a,1b). Three types of trichomes were observed under SEM of the leaf: macro, micro and glandular hairs (Figure1e). Macro hairs were located on silica cells over a thin vascular bundle, whereas micro and glandular hairs were located along the stomata cells or adjacent to motor cells (Figure1f). Very few macro hairs were observed on the lower (abaxial) surface of the leaf (Figure1g). On the glumes, hairs were distributed on the valleys between two swelled longitudinal veins (Figure1h).

Both the leaf and hull are glabrous and smooth in Yunnan Nuda Rice variety HMK (Figures1c and1d). Small bulges were present in place of macro hairs on the leaves (Figure1i), although glandular hairs and occasional micro hairs were still observed (Figure1j). There were no macro hairs on the lower (abaxial) surface (Figure1k). No hairs were observed on the surface of hulls (Figure1l).

### Genetic analysis and fine mapping of the NUDA locus

The genetic basis of the Nuda phenotype was analyzed. All F_1_ hybrids resulting from the cross between TN1 and a Yunnan Nuda Rice variety, HMK, exhibited a trichomous leaf phenotype. One-hundred and fifty-eight F_2_ individuals were screened and 120 trichomous leaf individuals and 38 glabrous individuals were identified. A three to one (*χ*^2^ = 0.076, *χ*^2^_0.05_ = 3.84) segregation ratio was determined. These results indicated that the Nuda phenotype was controlled by a single pair of recessive genes.

**Figure 2:**
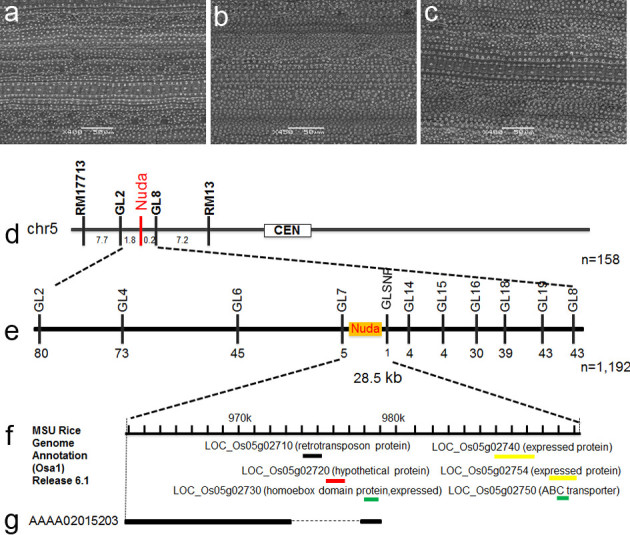
**Genetic analysis and fine mapping of** ***NUDA/GL-1.*** **a** SEM images of HMK leaf adaxial epidermis. **b** SEM images of Lemont leaf epidermis. **c** SEM images of HMK/Lemont F_1_ leaf epidermis. **d** Location of *NUDA* on rice chromosome 5 in 158 F_2_ plants. Numbers under the linkage map indicate genetic distance between adjacent markers. **e** High-resolution linkage map of the *NUDA* region produced with 1,192 F_2_ plants. The number of recombinants between markers is indicated under the linkage map. **f** Six putative genes were annotated within the 28.5 kb region according to MSU Rice Genome Annotation Release 6.1. **g** The large deletion in 9311 (Dotted line).

American rice varieties commonly exhibit a glabrous phenotype similar to that of Yunnan Nuda Rice. The *GL-1* gene conferring this phenotype has been mapped to the start of chromosome 5 (Chr. 5). To determine whether *NUDA* is allelic to *GL-1*, the HMK variety was crossed with the glabrous American variety, Lemont. All F_1_ plants exhibited a glabrous phenotype (Figure2a-c), which indicated that the *NUDA* locus is allelic to *GL-1*.

Simple sequence repeat (SSR) markers located at the start of Chr. 5 were used for the primary mapping of *NUDA*. Linkage analysis of 158 F_2_ individuals showed linkage of the RM17713 and RM13 markers with the *NUDA* locus at distances of 7.7 and 7.2 cM, respectively (Figure2d). The *NUDA* locus was mapped between markers GL2 and GL8 (Figure2d). A high-resolution genetic and physical map for the fine mapping of *NUDA* was constructed by the analysis of 11 newly developed markers, including 10 insertion/deletion (InDel) markers and one single nucleotide polymorphism (SNP) marker in 1,192 F_2_ Nuda individuals derived from the TN1 and HMK cross (Table1). The *NUDA* locus was mapped to a 28.5 kb interval between markers GL7 and GLSNP (Figure2e). Six annotated genes were identified in this defined interval based in P1 artificial chromosome (PAC) clone AP001111 from the Nipponbare variety. These were *LOC_Os05g02710* (retrotransposon protein), *LOC_Os05g02720*(hypothetical protein), *LOC_Os05g02730* (*WUSCHEL*-related homeobox 3B), *LOC_Os05g02740* (expressed protein), *LOC_Os05g02750* (membrane protein) and *LOC_Os05g02754* (expressed protein) (Figure2f).

**Table 1:** **Markers used for** ***NUDA/GL-1*** **gene mapping**

Marker name	Forward primer(5'–3’)	Reverse primer(5'–3’)	Marker type
RM17713	TTGTAACCACCAGCAGCAGGG	AGCAATGGTACAAATAGCCAAGC	SSR
GL2	AGTAAAATTGGATGTCATTTGGTA	CAAGAAAACTTCAGTACATTGGTG	InDel
GL4	CCGCTACGGAAGGGTAATAATG	AGTCGAAAGGATGGGAGGAGAA	InDel
GL6	TTATACTTTCATACGCACGATG	AACCTAGCTGATGGGTCTAGAT	InDel
GL7	GGGTTTGGGTGGTCCTCTC	CATGCACGCCGAGTAGCT	InDel
GLSNP	GCCGGTGATCGACAACGCCA	TGGGATCAGCTGAAAGTCTGTCCA	Direct sequence
GL14	GCCGTGCAACACGATATGG	GGCTTGTTTGGCGGTCAC	InDel
GL15	AGTGCCACAAATATAGCTCCG	CATTTTGATCTATTATAGTGATGATT	InDel
GL16	CACCGCTTTACGAACGCC	AGCAGCCACCTTCACGAG	InDel
GL18	TACCTGGGCGCATGAACA	CGGCAAGTTTTTGGGCTA	InDel
GL19	TTTTACTAGAAAAGATGCTCTGTT	CCACAATAAATTTCACATTAGC	InDel
GL8	TGTACATTCGATTACACGAGATAA	GCGAGAGGTGAGTAGGGCT	InDel
RM13	TCCAACATGGCAAGAGAGAG	TATCACATTCGATTCCAGCATG	SSR

### NUDA/GL-1 candidate genes

Genomic sequences of the candidate region defined by the markers GL7 and GLSNP were retrieved from the sequence of Nipponbare PAC clone P0699E04 (AP001111) and 9311 contig Ctg015203 (AAAA02015203). A large deletion including *LOC_Os05g02710* and *LOC_Os05g02720* in 9311 was confirmed by PCR and sequence analyses, indicating that these genes do not encode *NUDA/GL-1* in the pubescent Nipponbare and 9311 varieties (Figure2g).

The remaining four candidate genes were identified as follows: *LOC_Os05g02750* encodes a bacterial-type ATP binding cassette (ABC) transporter protein involved in efflux transport activity specific for UDP-glucose and aluminum tolerance in rice (Ma et al.[2009]); *LOC_Os05g02740* and *LOC_Os05g02754* encode expressed proteins with unknown functions; and *LOC_Os05g02730*, a *WUSCHEL*-like homeobox gene (*OsWOX3B*).

**Figure 3:**
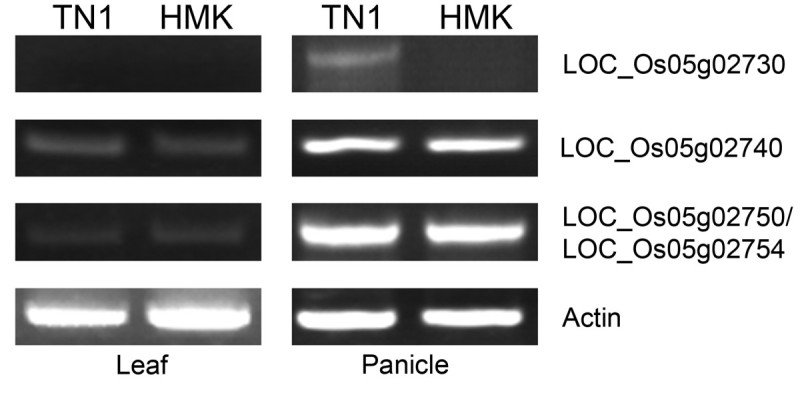
**RT-PCR expression analysis of four candidate genes in rice leaf and panicle.**

The sequences and expression patterns of these four genes were investigated to identify the *NUDA/GL-1* candidate gene. The sequences of these genes were identical to those of Nipponbare. Therefore, the expression patterns of these genes were further investigated in leaves and young panicles from HMK and TN1 varieties. A similar expression pattern of *LOC_Os05g02740* and *LOC_Os05g02750/ LOC_Os05g02754* was observed in both varieties. *LOC_Os05g02730* expression was detected in the panicles of TN1 but not HMK, although expression of this gene was not detected at vegetative stage in either variety (Figure3). These results implicated *LOC_Os05g02730* (*OsWOX3B*) as the *NUDA/GL1* candidate gene.

### RNAi and genetic complementation of the NUDA/GL-1 gene

**Figure 4:**
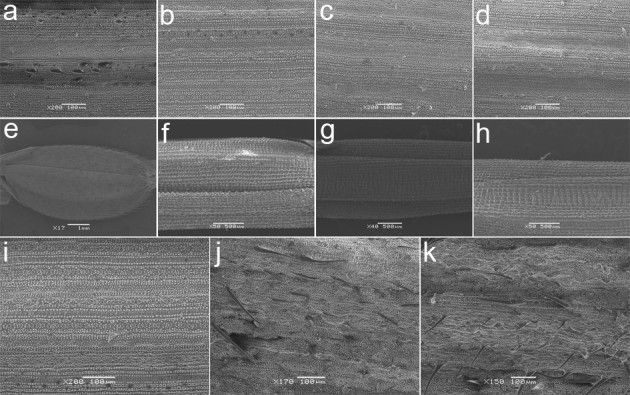
**SEM images of adaxial epidermis of leaf and grain.** **a** Nipponbare leaf **b** *OsWOX3/NUDA* RNAi line T1325 leaf, **c** T1326 leaf, **d** T1328 leaf, **e** Nipponbare grain, **f** *OsWOX3/NUDA* RNAi line T1325 grain, **g** T1326 grain, **h** T1328 grain, **i** leaf of HMK, **j** complementation transgenic line 1 leaf, **k** complementation transgenic line 2 leaf.

RNAi and genetic complementation analyses were performed to confirm that *OsWOX3B* corresponded to *NUDA/GL-1*. The transfer of an RNAi construct designed to repress *OsWOX3B* expression into Nipponbare resulted in transgenic lines that exhibited a glabrous phenotype in leaves (Figure4a-d) and hulls (Figure4e-h) from T0 generation. Furthermore, a 4.2 kb fragment including the promoter and coding region of *OsWOX3B* was cloned into pCAMBIA1301 and transferred into HMK. Pubescent transgenic rice plants with trichomes on the leaves were obtained, although the shapes of the trichomes were different and the alignments of the cells were disordered, which probably were due to the ectopic expression of *OsWOX3B* in transgenic plants (Figure4i-k). These results indicated that the *OsWOX3B* corresponded to the *NUDA/GL-1* gene and was responsible for the glabrous phenotype in HMK Nuda rice.

### NUDA/GL-1/OsWOX3B encodes a member of WOX3 subfamily

**Figure 5:**
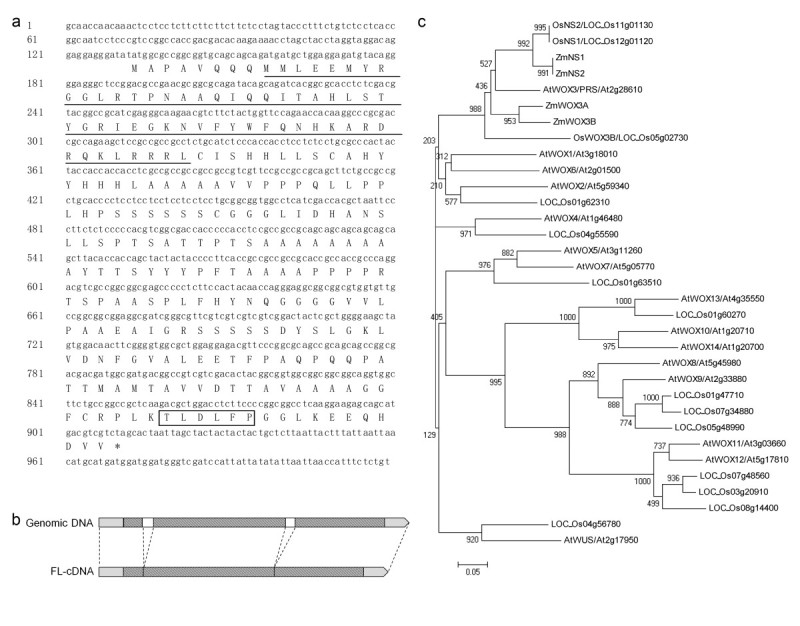
**NUDA/GL-1/OsWOX3B encodes a member of WOX3 subfamily.** **a** Sequences of cDNA and amino acid of *NUDA/GL-1/OsWOX3B*, the homeodomain is underlined, and the *WUSCHEL* domain is framed. **b** The splicing pattern of *NUDA/GL-1/OsWOX3B*, the open bar indicates the introns, the diagonal bar indicates the ORF, the gray bar indicates the UTRs. **c** Phylogenetic analysis of *WOX* genes. Neighbor-joining trees of WOX proteins from rice (Os), *Arabidopsis* (At) and maize (Zm). For the neighbor-joining tree, 1,000 boot-strap samples were generated to assess support for the inferred relationships.

Both the 5’ and 3’ ends of the *NUDA/GL-1/OsWOX3B* cDNA were obtained by the rapid amplification of cDNA ends (RACE) from the mRNA extracted from the panicles of the pubescent variety. The full length of *NUDA/GL-1/OsWOX3B* cDNA is 1019 bp, including an open reading frame with 780 bp, a 5’-untranslated region (UTR) with 132 bp and a 3’-UTR with 107 bp (Figure5a). By comparing the sequences of the full length cDNA and the relevant genomic region, two introns were identified (Figure5b). The putative protein contains a homeodomain (HD), which is a DNA-binding domain and a *WUSCHEL* domain, which indicated it belonged to the *WUSCHEL*-like homeobox (WOX) gene family (Figure5a).

Fourteen *OsWOX* genes and fifteen *AtWOX* genes were identified based on previous studies (Dai et al.[2007]; Haecker et al.[2004] Nardmann et al.[2007] Vandenbussche et al.[2009]Zhang et al.[2010]) and BLAST results in this study. BLAST searches of the NCBI Non-redundant Protein Sequences Database (http://blast.ncbi.nlm.nih.gov/) were performed using the homeodomain sequence of *AtWUS* (*At2g17950*) (Haecker et al.[2004]) as a query. Phylogenetic analysis of these genes and four maize *WOX* genes, which belong to the same subgroup of *OsWOX3,* was performed based on the sequences of the homeodomain regions. *OsWOX3B* was shown to be a homolog of *AtWOX3/PRS* (Figure5c). In contrast to *Arabidopsis*, three rice genes and four maize genes were identified in the *WOX3* subfamily. The phylogenetic tree map indicated that genes of *WOX3* subfamily were divided into several subgroups. *LOC_Os11g01130* (also known as *OsNS2* or *OsWOX3*) (Zhang et al.[2010]; Dai et al.[2007]), *LOC_Os12g01120* (also known as *OsNS* or *OsNS1*) (Nardmann et al.[2007]; Zhang et al.[2010]), *ZmNS1* and *ZmNS2* were tightly related and close to *AtWOX3/PRS*. *NUDA/GL-1/OsWOX3B* was in a separate subgroup (Figure5c).

**Figure 6:**
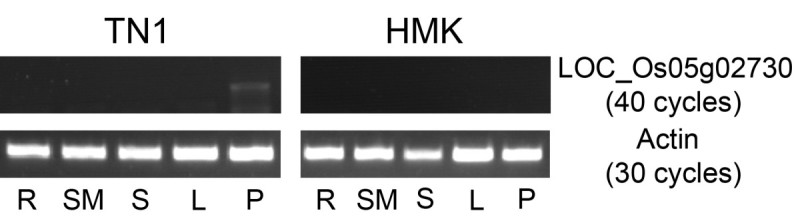
**RT-PCR of** ***OsWOX3B/NUDA/GL-1*** **in the root, SAM, leaf sheath, leaf blade and panicle from TN1 and HMK.** R, root; SM, SAM; S, leaf sheath; L, leaf blade.

The expression patterns of *NUDA/GL-1/OsWOX3B* in the root, shoot apical meristem (SAM), leaf sheath, leaf blade and panicle from the TN1 and HMK varieties were detected by RT-PCR. The expression of this gene could be detected in the panicle but not in the root, SAM, sheath or blade in pubescent rice TN1. In Nuda Rice HMK, no expression was detected in each tested tissue (Figure6).

### Trichome deficiency increases efficiency of grain packaging

**Figure 7:**
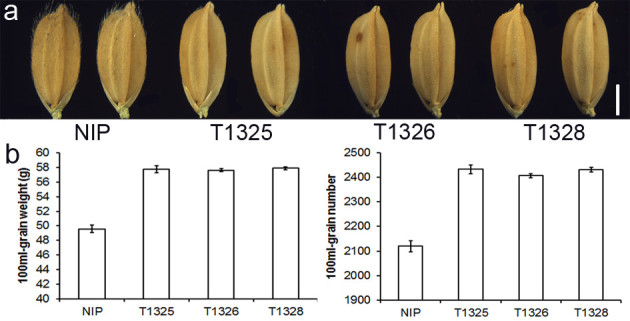
**Trichome deficiency increases efficiency of grain packaging.** **a** Grains of Nippare and three *NUDA/GL-1/OsWOX3B* RNAi transgenic lines (Scale bar: 2mm). **b** Packed dry grain numbers and weights of Nipponbare and three *OsWOX3B/NUDA/GL1* RNAi transgenic lines in 100 ml volume.

The agronomic traits of Nipponbare and three *OsWOX3B/NUDA/GL-1* RNAi transgenic lines were investigated. No significant differences were observed in terms of plant height, tiller number, specklet number per panicle, seed setting percentage and grain weight in RNAi lines compared with Nipponbare (Additional file 1: Table S1). These results indicated that *OsWOX3B/NUDA/GL-1* RNAi had no effect on these agronomic traits. Similarly, no significant differences were observed in grain size and shape, including grain length, grain width, grain thickness and ratio of length and width in RNAi lines compared with Nipponbare. These findings indicated that *OsWOX3B/NUDA/GL-1* RNAi did not affect the size and shape of grains (Figure7a,Additional file 2: Figure S1). However, the efficiency of grain packaging was increased in the three RNAi transgenic lines as measured by the number and weight of dried grains in a 100 ml volume (Nipponbare, 2,119.2 ± 23.0 grains weighing 49.6 ± 0.5 g; three *OsWOX3B/NUDA/GL-1* RNAi transgenic lines: 2,432.7 ± 18.4 grains weighing 57.8 ± 0.5 g; 2,406.5 ± 9.1 grains weighing 57.6 ± 0.2 g; and 2,431.9 ± 9.2 grains weighing 57.9 ± 0.2 g) (Figure7b).

## Discussion

The glabrous trait is beneficial for rice production due to the consequent reduction in dust pollution during grain harvesting, drying and processing. Furthermore, it has been observed that the bulk density of the glabrous rice varieties was higher (Rutger and Mackill[2001]). Theoretically, the lack of trichomes can make the glabrous kernels packed closer because the spaces occupied by the trichomes are released. It is clear that the size and shape of the grains also affect the grain packaging efficiency. In this study, the grain size and shape of *OsWOX3B* RNAi lines were similar with those of wild type plants. These strictly isogenic materials provided convincing evidence that lack of glum hair truly increased the efficiency of grain packaging. Glabrous rice varieties have been found in many locations worldwide and glabrous germplasm has been used to develop glabrous varieties. Studies suggest the existence of at least two loci that confer the glabrous phenotype in leaves and hulls although they have not been cloned. In this study, the *NUDA* locus of Yunnan Nuda Rice was demonstrated to be allelic to the *GL-1* locus of American glabrous varieties. Furthermore, the *NUDA/GL-1* gene was cloned by using a map-based approach and the locus was shown to encode a *WUSCHEL*-like homeobox gene (*OsWOX3B*) by RNAi and complementation transgenic studies.

Trichome is an excellent model system to study cell fate determination, cell cycle regulation, cell polarity and cell expansion in the model plant species, *Arabidopsis*. Recently, a number of genes that control the initiation and morphogenesis of trichomes have been identified using this model (Serna and Martin[2006]; Szymanski et al.[2000]), many of which encode several classes of transcriptional factors including MYB, WD-40, bHLH and HD-ZIP. However, experimental evidence has suggested that this model is limited to plants that belong to the Rosid division of the Eudicots (Serna and Martin[2006]). The *PALE ALEURONE COLOUR* (*PAC*) gene in maize that encodes a WD repeat protein can complement the glabrous phenotype of *ttg1* mutant in Arabidopsis. Interestingly, the *pac* mutant has no trichome phenotype (Carey et al.[2004]). In this study, a *WUSCHEL*-like homeobox (*WOX*) gene was identified that controlled trichrome development on the leaf and hull. These results suggested a separate pathway for trichome development in monocot plants and provided new evidence for the convergent evolution of trichome development pathways.

The *WOX* genes, which are specifically expressed in plants, form a large family that is a subgroup of the homodomain (HD)-containing transcription factors. WOX members characterized to date are involved in regulating diverse aspects of development. *LOC_Os11g01130* (*Wox3/OsNS2*) is involved in the direct repression of YAB3, which is required for rice leaf development (Dai et al.[2007]). *OsWOX11* is an auxin- and cytokinin-responsive gene and is required to activate shoot-borne crown root development (Zhao et al.[2009]). Ectopic expression of QHB affects normal shoot and leaf development (Kamiya et al.[2003]). In this study, a member of the *WOX3* subfamily was demonstrated to be involved in trichome development.

In addition to *OsNS2*, several other *WOX3* genes have been investigated. *AtWOX3/PRS* is expressed in the periphery of the shoot meristem and recruits founder cells from all meristem layers to form lateral domains of both vegetative and floral organs. Ectopic expression of PRS forms multicellular bulges on the stem and sepal ([Matsumoto and Okada 2001]). The maize duplicate genes narrow sheath 1 (*NS1*) and narrow sheath 2 (*NS2*) function in a lateral domain of shoot apical meristems (Nardmann et al.[2004]). Mutant leaves miss a large domain, including the leaf margin and mutant internodes are shortened on the marginal side of the stem (Nardmann et al.[2007]). *NS* and *PRS* show conserved function during the recruitment of organ founder cells from the lateral domain of plant meristems (Nardmann et al.[2004]). However, none of these genes are involved in trichome development. The presence of orthologs in monocots, such as *OsWOX3B* in rice, which root outside *NS/PRS* branching, indicated the occurrence of duplication after the separation of monocot and dicot species (Nardmann et al.[2007]). The expression patterns of duplicated no-*NS/PRS* branching paralogs were different from those of *NS/PRS* branching paralogs, which indicates subfunctionalization (Nardmann et al.[2007]). The new function identified in *WOX3* members outside *NS/PRS* branching in this study also supports the occurrence of subfunctionalization in this gene family.

In this study, the expression of *OsWOX3B* was only detected in the panicle of TN1. It is reported that *PRS* expresses only at a specific developmental stage ([Matsumoto and Okada 2001]). The negative detection of *OsWOX3B* in leaf in this study could also be due to the spatiotemporal expression of this gene. Interestingly, *OsWOX3B* expression was detected in the rice panicle of TN1 but not HMK rice. The transgenic *OsWOX3B* RNAi lines displayed a glabrous phenotype. These results suggested that *OsWOX3B* expression was suppressed in Yunnan Nuda Rice. However, no differences were identified in the coding or promoter sequences of this gene in comparisons of the HMK and pubescent Nipponbare varieties. It can be speculated that the glabrous phenotype of Yunnan Nuda rice results from epigenetic variations, such as DNA methylation.

Glabrous germplasms have been used to develop dust pollution free rice varieties. In this study, no differences were identified in the *OsWOX3B* gene sequences of pubescent rice and glabrous Yunnan Nuda rice, which would provide an advantageous functional molecular marker for breeding purposes. However, a dominantly inherited glabrous phenotype is obtained by RNAi suppression of *OsWOX3B*, thus indicating a novel future strategy for the development of glabrous rice, particularly glabrous hybrid rice.

## Conclusion

Yunnan Nuda Rice exhibited a typical glabrous phenotype. The Nuda phenotype was shown to be controlled by a pair of recessive genes allelic with the well-characterized *GL-1* locus. The *NUDA/GL-1* locus was fine mapped to a 28.5 kb region. A *WUSCHEL*-like homeobox gene (*OsWOX3B*) was confirmed as the candidate gene by RNAi and complementation analyses.

## Methods

### Plant materials

In this study, Yunnan Nuda Rice variety HMK was used to identify the *NUDA* gene. The American glabrous rice variety with *gl-1* gene Lemont was used for allelic tests. The pubescent indica variety TN1 was crossed with HMK to construct the mapping population. The pubescent variety Nipponbare was used as the receptor of *OsWOX3B* RNAi. All plants were grown in paddy fields in Shanghai, Hangzhou China during the summer and Hainan, China during the winter.

### Scanning electron microscope analysis

Leaves were dissected, fixed overnight at 4°C in FAA (formalin:glacial acetic:70% ethanol = 1:1:18) and dehydrated in a graded ethanol series (50, 70, 80, 90, 95 and 100%). Samples were critical-point-dried, mounted, sputter-coated with platinum and observed and photographed using a scanning electron microscope (JSM–6360LV; JEOL Ltd, Tokyo).

### DNA extraction and molecular marker analysis

Rice genomic DNA was extracted from fresh leaves according to the CTAB method ([Murray and Thompson 1980]). SSR markers information was obtained from Gramene (http://www.gramene.org). To construct a high-density linkage map for fine mapping in the target region, new insertion/deletion (InDel) markers and single nucleotide polymorphism (SNP) markers were developed according to the sequence differences between indica var. 93–11 and japonica var. Nipponbare (http://www.ncbi.nlm.nih.gov). Primers flanking the InDel and SNP polymorphisms were designed using the Primer Premier 5.0 program and tested on the parent varieties.

### Linkage map and gene mapping

Two newly developed markers and two polymorphic SSR markers (RM17713 and RM13) were used to construct a linkage map of *NUDA/GL-1* using Mapmaker/Exp 3.0 (Lander et al[1987]) on the basis of the 158-plant F_2_ population. The marker order and the genetic distance between every two adjacent markers were determined in the target region on chromosome 5. These informative molecular markers and newly developed markers were used for genotyping each plant of the F_2_ population to identify recombinants in the target region. The linkage relationship between markers and the *NUDA/GL-1* locus was analyzed. By assaying the recombinant events, the *NUDA/GL-1* locus was narrowed down to a 28.5 kb region on the AP001 111 PAC clone.

### RNA isolation, RT-PCR analysis and RACE

Total RNA was extracted from various rice tissues using EASYspin (Yuanpinghao, Tianjin). The extracted RNA was treated with RNase-free DNaseI (Frementas) to eliminate genomic DNA contamination according to the protocols recommended by the manufacturer.

First strand cDNA was synthesized from 2.5 μg total RNA using the RevertAid^TM^ First Strand cDNA Synthesis Kit (Fermentas, K1622). RT-PCR was performed with pairs of locus specific primers as follows:

*LOC_Os05g02730*, forward: 5’-CTTACACCACCAGCTACTACTACCC-3’, reverse, 5’-CTAGACGACGTCATGCTGCTCT-3’;

*LOC_Os05g02740*, forward: 5’-TGGGTTTCCTGCAAAACACT-3’, reverse 5’-CTACCGCCAGGCTTCTTGTA-3’;

*LOC_Os05g02750/ LOC_Os05g02754*, forward: 5’-TGTTCATCACCACGATCTGC-3’, reverse 5’-TGAAGAAGCTGAGGGAGGA-3’)

Rice *OsActin1* control gene, forward: 5’-TGCTATGTACGTCGCCATCCAG-3’, reverse, 5’-AATGAGTAACCACGCTCCGTCA-3’).

To obtain the full length-cDNA of *LOC_Os05g02730*, 5’ and 3’ RACE were undertaken by using 5’RACE System for Rapid Amplification of cDNA Ends Version 2.0 (Invitrogen, Catalog no.18374-058) and 3’-Full RACE Core Set Ver.2.0 (TaKaRa, Code: D314). The GSP1 primer for 5’ RACE is: 5’-CTAGACGACGTCATGCTGCTCT-3’. The GSP1 primer for 3’ RACE is:5’-CTTACACCACCAGCTACTACTACCC-3’.

### In silico analysis of NUDA/GL-1/OsWOX3B protein

Conserved domain in NUDA/GL-1/OsWOX3B protein was searched in NCBI (http://www.ncbi.nlm.nih.gov/Structure/cdd/wrpsb.cgi). Blast search parameters were set as the default.

To search the *WOX* genes in Arabidopsis and rice, BLAST was performed using the Blastp program to search the NCBI Non-redundant Protein Sequences Database (http://blast.ncbi.nlm.nih.gov/) using the homeodomain sequence of *AtWUS* as a BLAST query. The collected amino acid sequences were aligned using the CLUSTALW program (http://www.ddbj.nig.ac.jp/search/clustalw-j.html) with standard parameters. A phylogenetic tree was generated with the neighbor-joining method and 1,000 bootstrap samples were generated to assess support for the inferred relationships.

### Vector construction and transformation

*LOC_Os05g02730* was amplified with its 2 kb native promoter from Nipponbare genome DNA using primers 02730-clone-f (5’-GGTGTAACGTCTGCCCAAGT-3’) and 02730-clone-r (5’-CATTCCATCCATACGCTTGA-3’). The 4.2 kb PCR product was cloned into the pMD18T (TaKaRa) vector and sequenced. The verified clone was digested with *Hind* III and *Eco* RI and the DNA fragment was cloned into pCAMBIA1301 (http://www.cambia.org). To construct the *LOC_Os05g02730* RNAi vector, the third exon was amplified using primer 02730-RNAi-f (5’-GGGGACAAGTTTGTACAAAAAAGCAGGCTTCGGAGGCGGCGGCGTGGTGT-3’) and 02730-RNAi-r (5’-GGGGACCACTTTGTACAAGAAAGCTGGGTCCTAGACGACGTCATGCTGCT-3’) and the fragment was cloned into the gateway RNAi vector pANDA35HK (Miki et al.[2005]) (http://www.invitrogen.com/site/us/en/home/Products-and-Services/Applications/Cloning/Gateway-Cloning/GatewayC-Misc/Protocols.html#bp). The complementary and RNAi vectors were introduced into HMK and Nipponbare, respectively, by *Agrobacterium tumefaciens*-mediated transformation under selection with hygromycin at 50 mg/l ([Toki 1997]). The RNAi lines were verified with primers Guslinker-f (5’-CATGAAGATGCGGACTTACG-3’) and Guslinker-r (5’-ATCCACGCCGTATTCGG-3’).

### Agronomic traits and evaluation of efficiency of grain packaging

The agronomic traits of the Nipponbare and the homozygous T2 generation of three independent *OsWOX3/NUDA/GL-1* RNAi transgenic lines were individually surveyed during the summer of 2011 in Songjiang, Shanghai. Factors that included plant height, tillering number, grains per panicle, seed setting rate and 1,000-grain weight (g) were measured. The packaging efficiencies of the Nipponbare and the three RNAi transgenic lines were evaluated by measuring the number and weight of dried grains (about 10% of seed moisture contents) in 100 ml volume. Grain was harvested from four plants of each line. Mean values were compared and analyzed using a t-test.

## Misc

Honglei Zhang and Kun Wu contributed equally to this work.

## Electronic supplementary material

Additional file 1:**Table S1.** Evaluation of agronomic traits of Nipponbare and Nuda RNAi transgenic lines. The agronomic traits of the Nipponbare and three *OsWOX3/NUDA/GL1* RNAi transgenic lines were evaluated by measurements of the plant height, tillering number, grains per panicle, seed setting rate, and 1,000-grain weight. (g). (DOC 30 kb) (DOC 30 KB)

Additional file 2:**Figure S1.** The size and shape of the grains of Nipponbare and Nuda RNAi transgenic lines. a Grain length of Nipponbare and Nuda RNAi transgenic lines. b Grain thickness of Nipponbare and Nuda RNAi transgenic lines. c Grain width of Nipponbare and Nuda RNAi transgenic lines. d The grain length/width ratio of Nipponbare and Nuda RNAi transgenic lines. (TIFF 440 KB)

Below are the links to the authors’ original submitted files for images.

Authors’ original file for figure 1

Authors’ original file for figure 2

Authors’ original file for figure 3

Authors’ original file for figure 4

Authors’ original file for figure 5

Authors’ original file for figure 6

Authors’ original file for figure 7

## References

[CR1] Carey CC, Strahle JT, Selinger DA, Chandler VL (2004). Mutations in the pale aleurone color1 regulatory gene of the Zea mays anthocyanin pathway have distinct phenotypes relative to the functionally similar TRANSPARENT TESTA GLABRA1 gene in Arabidopsis thaliana. Plant Cell.

[CR2] Dai M, Hu Y, Zhao Y, Liu H, Zhou DX (2007). A WUSCHEL-LIKE HOMEOBOX gene Represses a YABBY gene expression required for rice leaf development. Plant Physiol.

[CR3] Foster KW, Rutger JN (1978). Independent segregation of semi-dwarfing genes and a gene for pubescence in rice. J Hered.

[CR4] Gibson PT, Maiti RK (1983). Trichomes in segregating generations of Sorghum matings.1. Inheritance of presence and density. Crop Sci.

[CR5] Haecker A, Gross-Hardt R, Geiges B, Sarkar A, Breuninger H, Herrmann M, Laux T (2004). Expression dynamics of WOX genes mark cell fate decisions during early embryonic patterning in Arabidopsis thaliana. Development.

[CR6] Hulskamp M, Schnittger A (1998). Spatial regulation of trichome formation in Arabidopsis thaliana. Semin Cell Dev Biol.

[CR7] Hulskamp M (2004). Plant trichomes: a model for cell differentiation. Nat Rev Mol Cell Biol.

[CR8] Johnson CS, Kolevski B, Smyth DR (2002). TRANSPARENT TESTA GLABRA2, a trichome and seed coat development gene of Arabidopsis, encodes a WRKY transcription factor. Plant Cell.

[CR9] Kamiya N, Nagasaki H, Morikami A, Sato Y, Matsuoka M (2003). Isolation and characterization of a rice WUSCHEL-type homeobox gene that is specifically expressed in the central cells of a quiescent center in the root apical meristem. Plant J.

[CR10] Karkkainen K, Agren J (2002). Genetic basis of trichome production in Arabidopsis lyrata. Hereditas.

[CR11] Kirik V, Simon M, Wester K, Schiefelbein J, Hulskamp M (2004). ENHANCER of TRY and CPC 2 (ETC2) reveals redundancy in the region-specific control of trichome development of Arabidopsis. Plant Mol Biol.

[CR12] Kumar KA, Andrews DJ (1993). Genetics of qualitative traits in pearl-millet - a Review. Crop Sci.

[CR13] Lander ES, Green P, Abrahanson J, Barlow A, Daly MJ, Lincon SE, Newburg L (1987). MAPMAKER: an interactive computing package for constructing primary genetic linkages of experimental and natural populations. Genomics.

[CR14] Larkin JC, Oppenheimer DG, Pollock S, Marks MD (1993). Arabidopsis GLABROUS1 gene requires downstream sequences for function. Plant Cell.

[CR15] Leisle D (1974). Genetics of leaf pubescence in wheat. Crop Sci.

[CR16] Li W, Wu J, Weng S, Zhang D, Zhang Y, Shi C (2010). Characterization and fine mapping of the glabrous leaf and hull mutants (gl1) in rice (Oryza sativa L.). Plant Cell Rep.

[CR17] Lim HH, Domala Z, Joginder S, Lee SH, Lim CS, Abu Bakar CM (1984). Rice millers' syndrome: a preliminary report. Br J Ind Med.

[CR18] Lloyd AM, Payne CT, Zhang F (2000). GL3 encodes a bHLH protein that regulates trichome development in arabidopsis through interaction with GL1 and TTG1. Genetics.

[CR19] Ma JF, Huang CF, Yamaji N, Mitani N, Yano M, Nagamura Y (2009). A bacterial-type ABC transporter is involved in aluminum tolerance in rice. Plant Cell.

[CR20] Marks MD (1997). Molecular genetic analysis of trichome development in Arabidopsis. Annu Rev Plant Physiol Plant Mol Biol.

[CR21] Matsumoto N, Okada K (2001). A homeobox gene, PRESSED FLOWER, regulates lateral axis-dependent development of Arabidopsis flowers. Genes Dev.

[CR22] Miki D, Itoh R, Shimamoto K (2005). RNA silencing of single and multiple members in a gene family of rice. Plant Physiol.

[CR23] Moose SP, Lauter N, Carlson SR (2004). The maize macrohairless1 locus specifically promotes leaf blade macrohair initiation and responds to factors regulating leaf identity. Genetics.

[CR24] Murray MG, Thompson WF (1980). Rapid isolation of high molecular weight plant DNA. Nucleic Acids Res.

[CR25] Nagao S, Takahashi M, Kioshita T (1960). Genetical studies on rice plant, XXV: Inheritance of three morphological characters, pubescence of leaves and floral glumes, and deformation of empty glumes. Journal of the Faculty of Agriculture, Hokkaido University.

[CR26] Nardmann J, Ji J, Werr W, Scanlon MJ (2004). The maize duplicate genes narrow sheath1 and narrow sheath2 encode a conserved homeobox gene function in a lateral domain of shoot apical meristems. Development.

[CR27] Nardmann J, Zimmermann R, Durantini D, Kranz E, Werr W (2007). WOX gene phylogeny in Poaceae: a comparative approach addressing leaf and embryo development. Mol Biol Evol.

[CR28] Oppenheimer DG, Herman PL, Sivakumaran S, Esch J, Marks MD (1991). A myb gene required for leaf trichome differentiation in Arabidopsis is expressed in stipules. Cell.

[CR29] Payne CT, Zhang F, Lloyd AM (2000). GL3 encodes a bHLH protein that regulates trichome development in arabidopsis through interaction with GL1 and TTG1. Genetics.

[CR30] Qin F, Zhang D, Lin X, Xie Y (1997). Classification of Yunnan Nuda compatible varieties. J Huazhong Agricultural University.

[CR31] Rutger JN, Mackill DJ (2001). Application of Mendelian genetics in rice breeding. Rice Genetics IV, Proceedings of the Fourth International Rice Genetics Symposium, 22–27 October 2000, Los Baños, Philippines.

[CR32] Sarkarung S, Collins F (1977). Inheritance of leaf pubescence in oats. AgronAbstr.

[CR33] Sato K, Takeda K (1992). Genetic analysis of large trichomes on the barley leaf blade. Barley Genet Newsl.

[CR34] Scanlon MJ, Schneeberger RG, Freeling M (1996). The maize mutant narrow sheath fails to establish leaf margin identity in a meristematic domain. Development.

[CR35] Schellmann S, Schnittger A, Kirik V, Wada T, Okada K, Beermann A, Thumfahrt J, Jurgens G, Hulskamp M (2002). TRIPTYCHON and CAPRICE mediate lateral inhibition during trichome and root hair patterning in Arabidopsis. EMBO J.

[CR36] Serna L, Martin C (2006). Trichomes: different regulatory networks lead to convergent structures. Trends Plant Sci.

[CR37] Southwood R, Juniper B, Southwood R (1986). Plant surfaces and insects - an overview. Insects and the plant surface.

[CR38] Szymanski DB, Lloyd AM, Marks MD (2000). Progress in the molecular genetic analysis of trichome initiation and morphogenesis in Arabidopsis. Trends Plant Sci.

[CR39] Toki S (1997). Rapid and efficient Agrobacterium-mediated transformation in rice. Plant Mol Biol Rep.

[CR40] Vandenbussche M, Horstman A, Zethof J, Koes R, Rijpkema AS, Gerats T (2009). Differential recruitment of WOX transcription factors for lateral development and organ fusion in Petunia and Arabidopsis. Plant Cell.

[CR41] Walker AR, Davison PA, Bolognesi-Winfield AC, James CM, Srinivasan N, Blundell TL, Eschc JJ, Marksc MD, Graya JC (1999). The TRANSPARENT TESTA GLABRA1 locus, which regulates trichome differentiation and anthocyanin biosynthesis in Arabidopsis, encodes a WD40 repeat protein. Plant Cell.

[CR42] Wang D, Sun S, Fa G, Lu X, Li Z, Ren G (2009). Mapping a rice glabrous gene using simple sequence repeat markers. Rice Sci.

[CR43] Werker E (2000). Trichome diversity and development. Adv Bot Res.

[CR44] Yu ZH, Mccouch SR, Kinoshita T, Sato S, Tanksley SD (1995). Association of morphological and RFLP markers in rice (Oryza-Sativa L). Genome.

[CR45] Zhang X, Zong J, Liu J, Yin J, Zhang D (2010). Genome-wide analysis of WOX gene family in rice, sorghum, maize, Arabidopsis and poplar. J Integr Plant Biol.

[CR46] Zhao Y, Hu Y, Dai M, Huang L, Zhou DX (2009). The WUSCHEL-related homeobox gene WOX11 is required to activate shoot-borne crown root development in rice. Plant Cell.

