# Supplementary material for: A WUSCHEL-like homeobox gene, OsWOX3B responses to NUDA/GL-1 locus in rice
Source: Rice (N Y). 2012 Oct 3;5:30. doi: 10.1186/1939-8433-5-30 (PMC5520835; doi:10.1186/1939-8433-5-30)
Supplement: Supplementary file 1 — Additional file 1:Table S1. Evaluation of agronomic traits of Nipponbare and Nuda RNAi transgenic lines. The agronomic traits of the Nipponbare and three OsWOX3/NUDA/GL1 RNAi transgenic lines were evaluated by measurements of the plant height, tillering number, grains per panicle, seed setting rate, and 1,000-grain weight. (g). (DOC 30 kb) (DOC 30 KB) [file 12284_2012_22_MOESM1_ESM.doc]

| Lines | Plant Height  (cm) | Tillering number | Grains per panicle | Seed setting rate  (%) | 1000-grain weight (g) |
| --- | --- | --- | --- | --- | --- |
| NIP | 79.6±3.5 | 25.8±6.6 | 71.0±18.9 | 77.3±4.5 | 23.4±0.1 |
| T1325 | 78.3±4.3 | 23.0±8.1 | 69.5±10.0 | 76.9±7.1 | 23.7±0.2 |
| T1326 | 74.4±2.0 | 23.0±6.7 | 74.6±14.0 | 75.9±7.6 | 23.9±0.1 |
| T1328 | 76.4±5.5 | 21.5±3.1 | 68.4±19.9 | 83.2±7.4 | 23.8±0.2 |

**Table S1**. Evaluation of agronomic traits of Nipponbare and Nuda RNAi transgenic lines.

The agronomic traits of the Nipponbare and three *OsWOX3/NUDA/GL1* RNAi transgenic lines were evaluated by measurements of the plant height, tillering number, grains per panicle, seed setting rate, and 1,000-grain weight (g).
