# Supplementary figures and images for: A WUSCHEL-like homeobox gene, OsWOX3B responses to NUDA/GL-1 locus in rice
Source: Rice (N Y). 2012 Oct 3;5:30. doi: 10.1186/1939-8433-5-30 (PMC5520835; doi:10.1186/1939-8433-5-30)

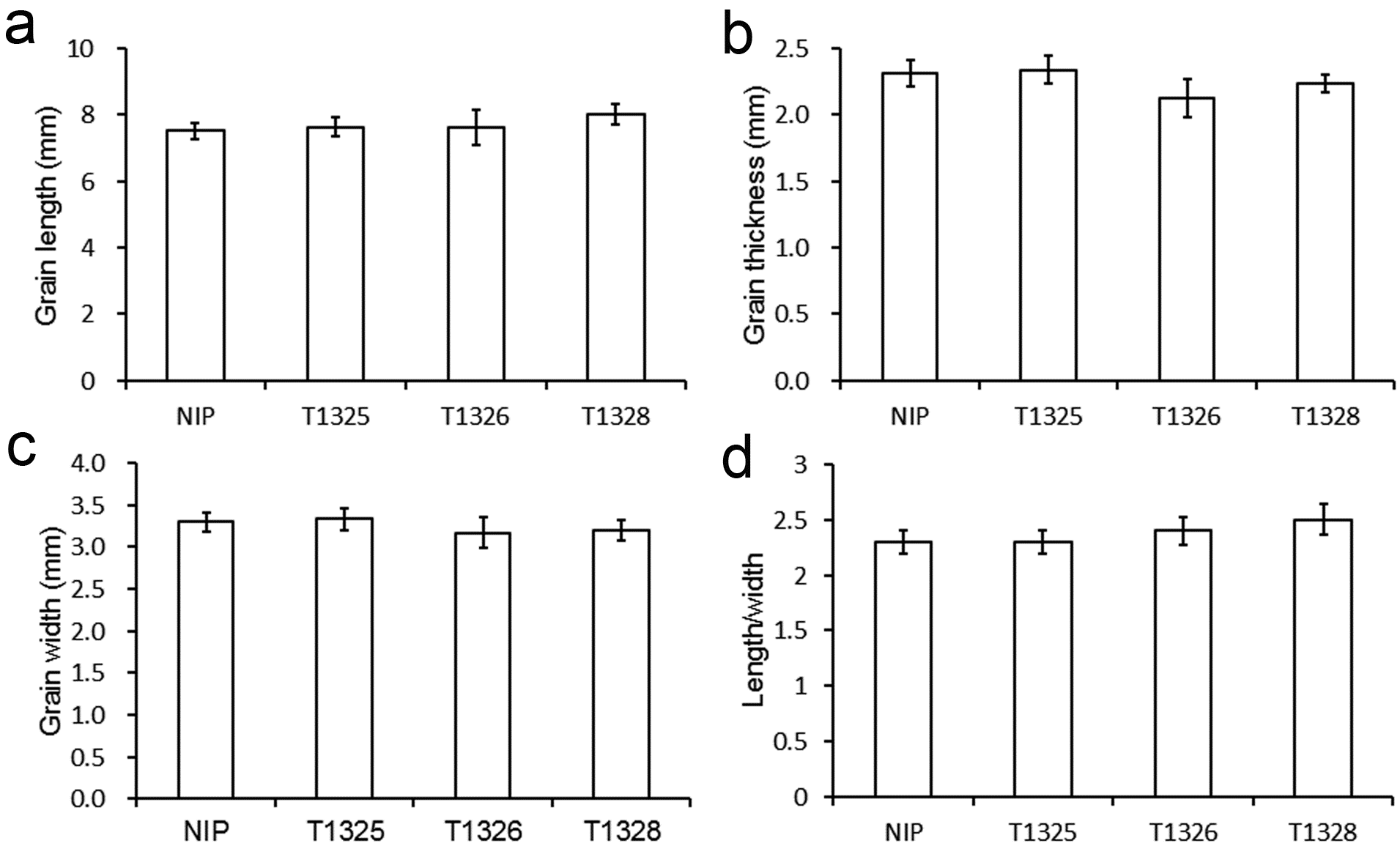

Supplement: Supplementary file 2 — Additional file 2:Figure S1. The size and shape of the grains of Nipponbare and Nuda RNAi transgenic lines. a Grain length of Nipponbare and Nuda RNAi transgenic lines. b Grain thickness of Nipponbare and Nuda RNAi transgenic lines. c Grain width of Nipponbare and Nuda RNAi transgenic lines. d The grain length/width ratio of Nipponbare and Nuda RNAi transgenic lines. (TIFF 440 KB) [file 12284_2012_22_MOESM2_ESM.tiff]

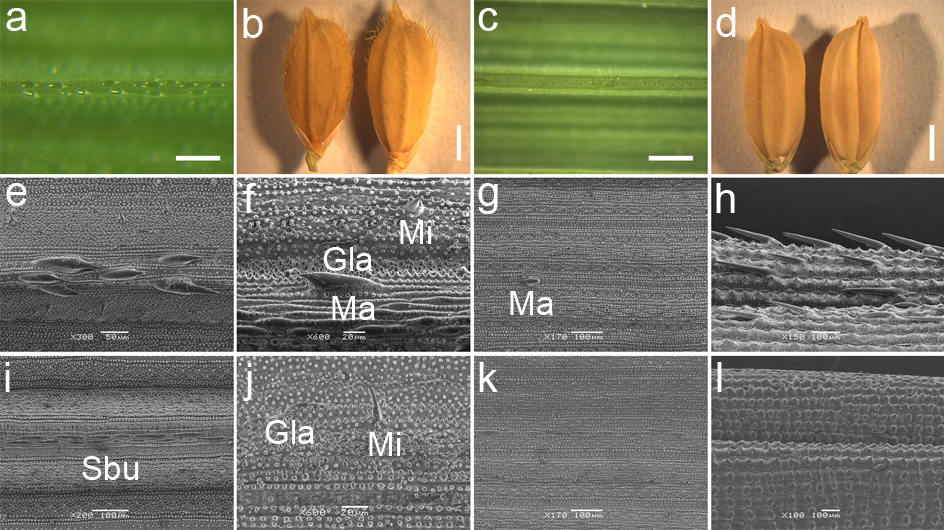

Supplement: Supplementary file 3 — Authors’ original file for figure 1 [file 12284_2012_22_MOESM3_ESM.tiff]

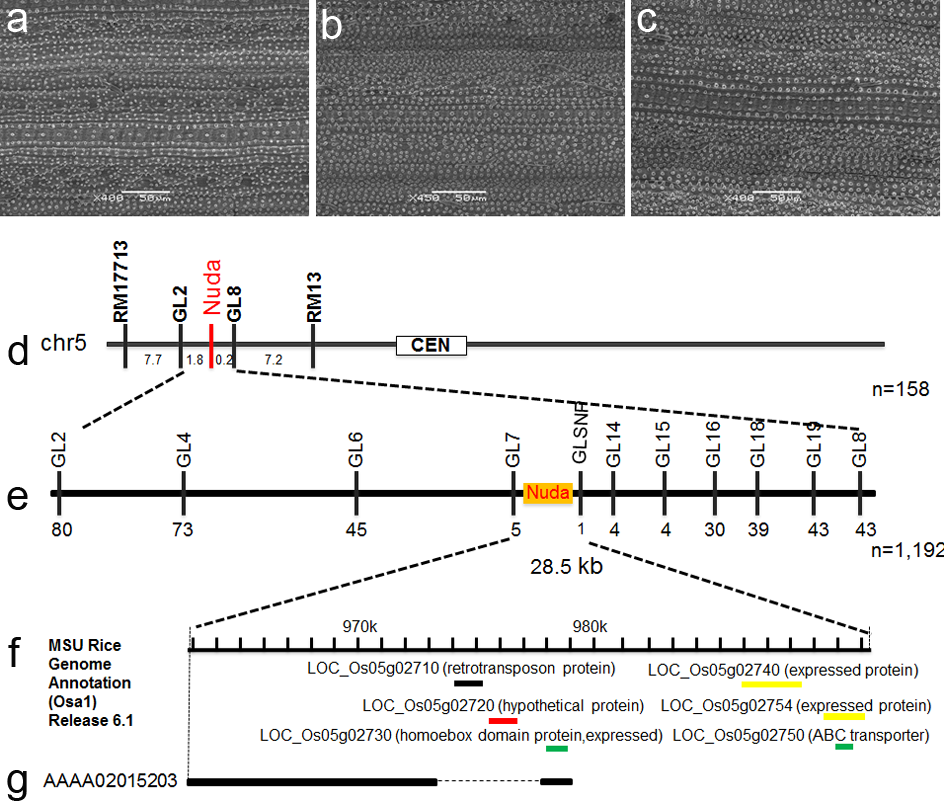

Supplement: Supplementary file 4 — Authors’ original file for figure 2 [file 12284_2012_22_MOESM4_ESM.tiff]

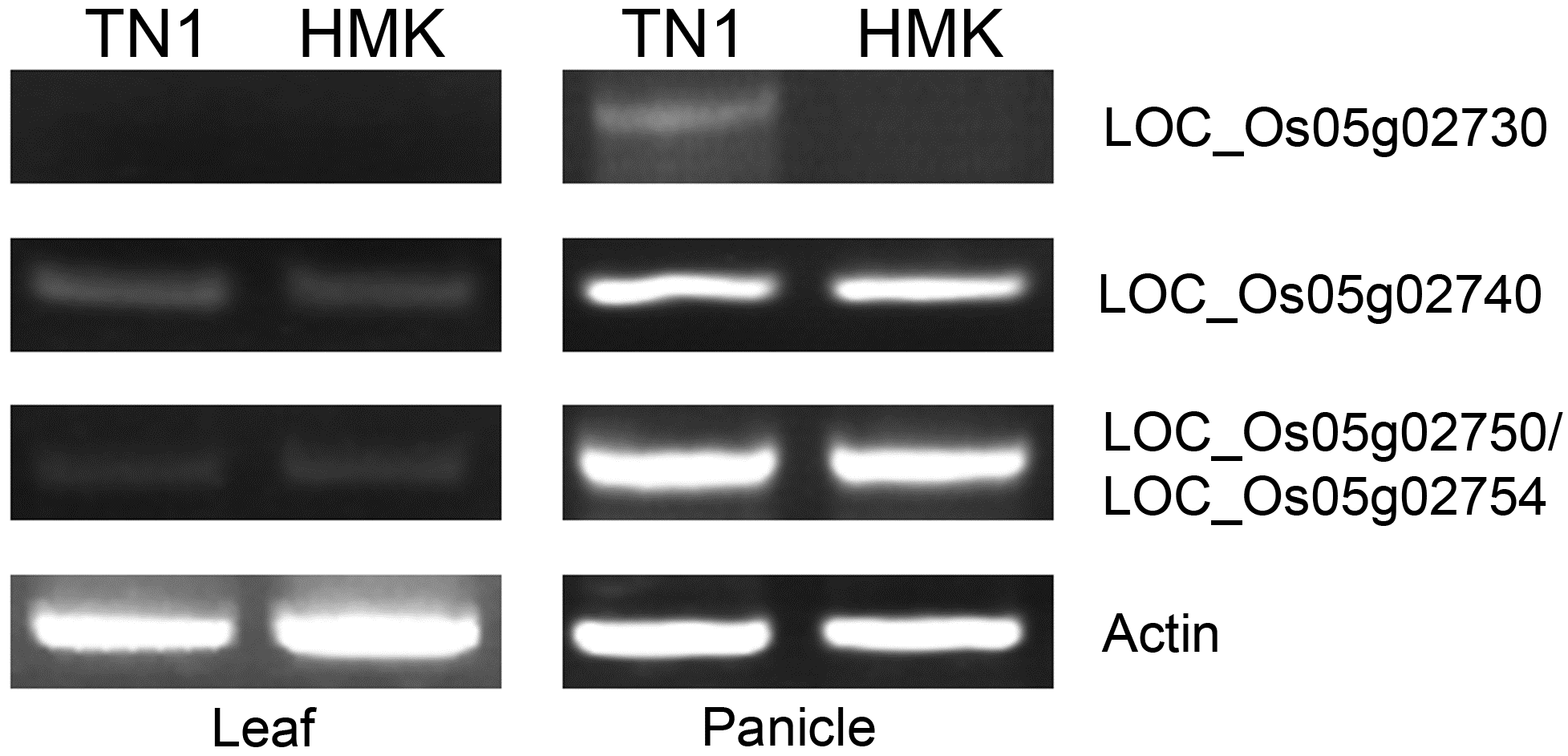

Supplement: Supplementary file 5 — Authors’ original file for figure 3 [file 12284_2012_22_MOESM5_ESM.tiff]

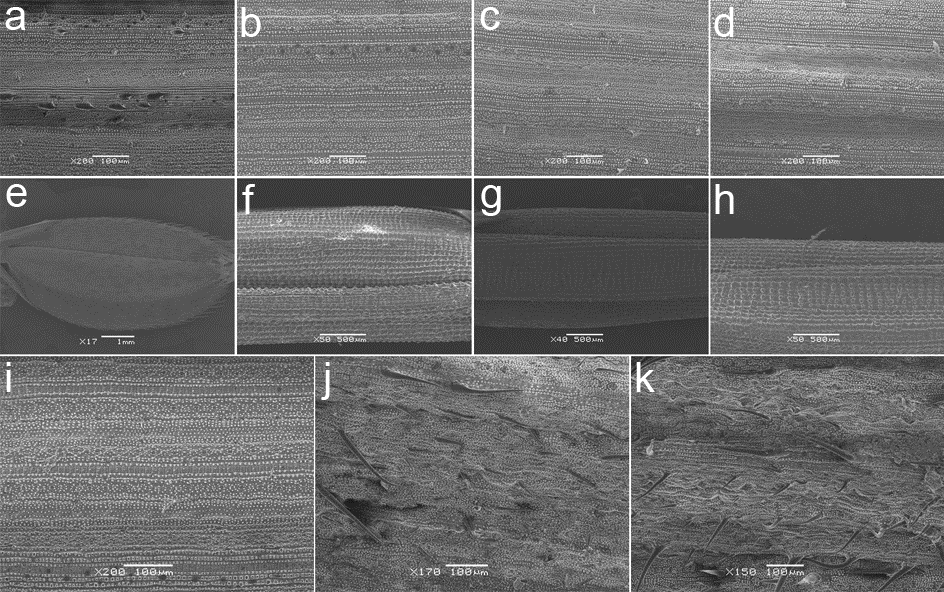

Supplement: Supplementary file 6 — Authors’ original file for figure 4 [file 12284_2012_22_MOESM6_ESM.tiff]

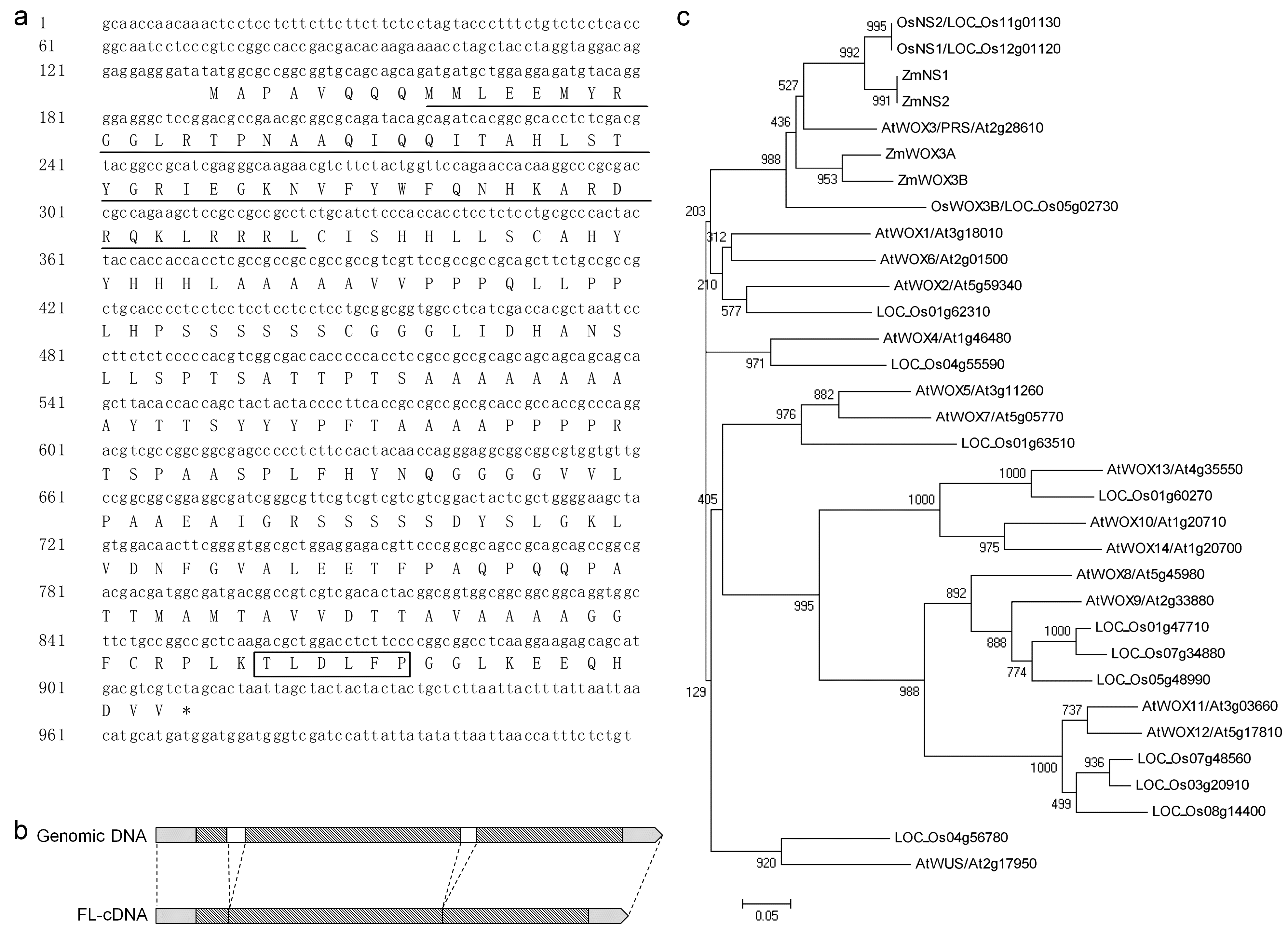

Supplement: Supplementary file 7 — Authors’ original file for figure 5 [file 12284_2012_22_MOESM7_ESM.tiff]

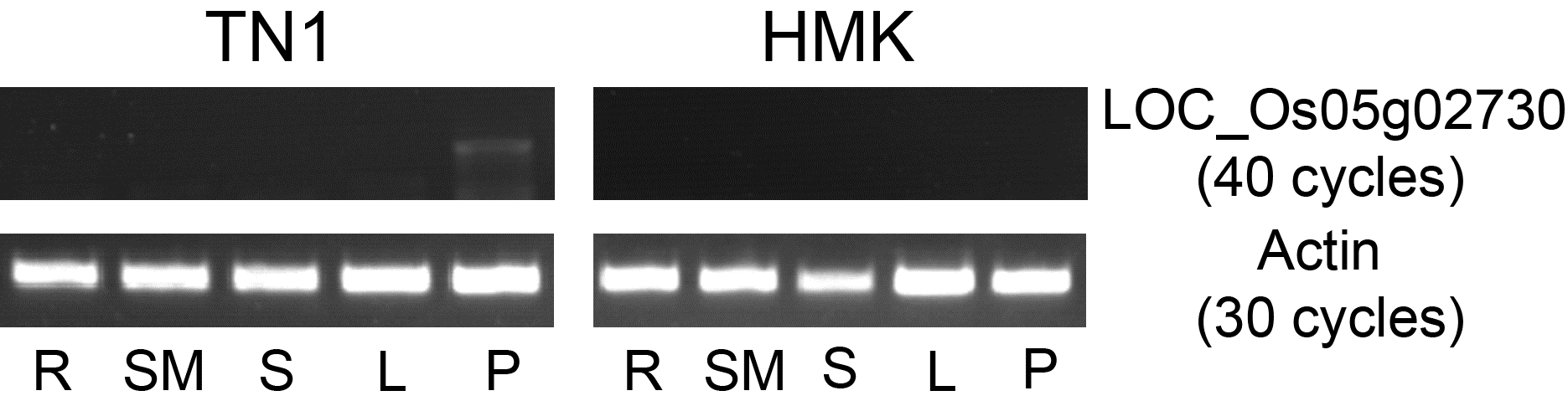

Supplement: Supplementary file 8 — Authors’ original file for figure 6 [file 12284_2012_22_MOESM8_ESM.tiff]

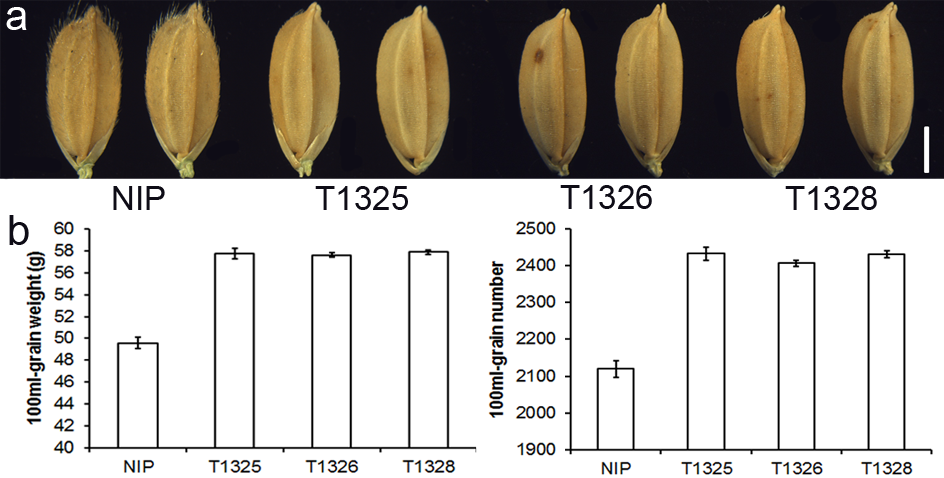

Supplement: Supplementary file 9 — Authors’ original file for figure 7 [file 12284_2012_22_MOESM9_ESM.tiff]
